# The incidence, characteristics, and complications of pregnant women who delivered stillbirths under different child policies in central China

**DOI:** 10.3389/fpubh.2025.1635120

**Published:** 2025-10-07

**Authors:** Lili Xiong, Donghua Xie, Qingyun Jiang, Junqun Fang

**Affiliations:** Hunan Province Maternal and Children Care Hospital, Changsha, China

**Keywords:** stillbirth, fertility policy, incidence, maternal comorbidities, risk factors

## Abstract

**Background:**

China’s evolving fertility policies (one-child to three-child) have shaped maternal and neonatal outcomes, but specific gaps in stillbirth epidemiology during policy transitions.

**Methods:**

This retrospective cohort study analyzed 721,860 singleton pregnancies in 2011–2023, from 18 maternal near-miss surveillance hospitals in Hunan. Stillbirth rates were assessed across four policy periods: one-child (2011–2013), partial two-child (2013–2015), universal two-child (2016–2020), and three-child (2021–2023). Multivariable logistic regression identified risk factors, adjusting for fertility policy period, maternal demographics and maternal comorbidities. Trends over time were analyzed using segmented regression models.

**Results:**

The overall stillbirth rate was 7.02‰ (95% confidence interval [CI]: 6.82–7.21), declining significantly from 9.62‰ during the one-child policy to 5.73‰ (95%CI: 5.25–6.23) under the three-child policy (*t* = −4.22, *p* < 0.01). Key risk factors included maternal age < 24 years (adjusted odds ratio [aOR] = 1.77, 95%CI:1.63–1.92), multiparity (aOR = 1.27–2.82. *p* < 0.01), non-rural hospital delivery (aOR = 4.00–11.13, *p* < 0.01), education ≤9 years (aOR = 1.51–2.20, *p* < 0.01), not being married (aOR = 2.92–5.60, *p* < 0.01), and comorbidities: severe preeclampsia (aOR = 3.80, 95%CI: 3.36–4.29), chronic hypertension (aOR = 2.67, 95%CI: 2.09–3.37), placental abruption (aOR = 5.06, 95%CI: 4.11–6.16), and placenta previa (aOR = 1.55, 95%CI: 1.29–1.84). Paradoxically, prenatal diabetes was associated with reduced stillbirth risk (aOR = 0.86, 95%CI: 0.77–0.95). Temporal shifts revealed elevated stillbirth rates among advanced-age mothers pre-2016 versus rising rates in women <24 years post-policy liberalization. Only the partial two-child policy period (aOR = 1.15, 95%CI: 1.05–1.25) was associated with the risk of stillbirth.

**Conclusion:**

China’s fertility policy transitions correlate with dynamic stillbirth epidemiology, emphasizing age- and parity-specific vulnerabilities. Targeted interventions for high-risk subgroups, especially younger, less well-educated, multiparous women, and those with hypertensive or placental disorders, are critical amid ongoing implementation of the three-child policy.

## Background

The definition of stillbirth varies among countries and regions around the world. The World Health Organization (WHO) defines it as fetal death as occurring at ≥28 weeks’ gestation, with birth weight ≥1,000 g or body length ≥35 cm (1). Contrastingly, the American College of Obstetricians and Gynecologists (ACOG) uses a lower viability threshold, classifying stillbirths as fetal demise at ≥20 weeks’ gestation (or undetermined gestational age) with birth weight ≥350 g (2). Other major developed economies show further variation: Australia and European nations typically use either 20-week gestational criteria (24 weeks in certain jurisdictions) or 500 g weight thresholds (3). The definition of stillbirths in China is consistent with the WHO definition.

Stillbirth is a significant component of perinatal mortality and a primary cause of medical disputes in healthcare institutions. It is also a crucial indicator of the quality and accessibility of perinatal care, particularly antenatal and intrapartum care (4). Despite sharing 72% of modifiable risk factors with maternal and neonatal mortality (including hypertensive disorders, infections, and placental insufficiency) (5), stillbirth reduction lags markedly in global health agendas, evidenced by an annual decline of a mere 2.3% since 2000, compared with reduction of 2.9% in neonatal mortality and 4.3% in mortality of children under 5 years old (6). This stagnation translates to preventable losses of 2 million viable fetuses annually. The United Nations has therefore explicitly included stillbirth prevention in its Global Strategy (2016–2030) alongside maternal and child mortality, through the landmark commitment to “end preventable stillbirths” (7). However, China lacks standardized integration of stillbirth metrics into core maternal and neonatal quality indicators, such as maternal and neonatal mortality rates, reflecting insufficient focus on this problem. Current research on stillbirth rate there remains fragmented, limited to Zhu’s national report (8, 9), and Shenzhen’s regional reports (10). Hunan Province-a demographic epicenter with 66 million residents-exemplifies this data desert, with no provincial-level stillbirth epidemiology information.

Global stillbirth surveillance shows significant epidemiological disparities, with an estimated 2 million late-gestation (≥28 weeks) fetal deaths annually (13.9‰ of total births) (11). When applying the 20-week gestational threshold, this increases to over 4 million deaths worldwide (4). Notably, 98% of stillbirths occur in low- and middle-income countries, with China ranking fifth in absolute numbers globally (5, 12). China’s stillbirth epidemiology shows dynamic temporal patterns influenced by evolving fertility regulations. National research data shows a prevalence of 8.8‰(≥28 weeks) during 2012–2014 (9), 13.2‰ (≥24 weeks) in 2015–2016 (8), and 5.5‰ (≥28 weeks) by 2019 (13). The prevalence of stillbirths may be linked to the effects of child birth policy. China introduced the one-child policy in 1978 (14), the partial two-child policy in November 2013 (15), and the universal two-child policy in October 2015 (16). However, the fertility rate in China has been declining every year since 2016. Since 2021, the Chinese government and many regions have introduced policies to encourage people to have second and third children (17) and China implemented a full three-child policy on May 31, 2021 (18).

China has accumulated substantial research evidence on the relationship between fertility policy adjustments and maternal and neonatal health outcomes. Investigations have primarily focused on two dimensions: maternal health aspects, including fertility intentions (19), second-time mothers (20), cesarean section rates (21), obstetric infection (22), uterine rupture (23), the characteristics of pregnancy and delivery (24), and perinatal outcomes, including birth defects (25–27), gender equity (17), early-term birth (28) and so on. Internationally, few studies have examined temporal changes in stillbirth rates, especially specific gaps in stillbirth epidemiology during policy transitions.

It is therefore both feasible and important to study the incidence of stillbirths and the characteristics of pregnancies that ended in stillbirths under different fertility policies. This will provide a reference for the incidence of stillbirths in central provinces of China, examine the causes of stillbirths, and demonstrate changes in the characteristics of stillbirths in central regions.

## Materials and methods

### Study design and data sources

This retrospective cohort study was conducted across 18 maternal near miss (MNM) surveillance hospitals in 14 cities within Hunan province, which aimed to enumerate all maternal deaths and near misses(women who nearly died from a severe complication of pregnancy or delivery) using the same approach as that proposed by WHO (29). The study population was pregnant or post-partum women admitted to obstetrics departments, with data collection spanning from hospital admission through to discharge. Data were systematically collected using the standardized “Maternal Case Survey Form” designed by the China Office of Maternal and Child Health Monitoring. This includes maternal characteristics, maternal and perinatal health, and pregnancy-related comorbidities and complications. The surveillance protocol incorporated three-tier quality control measures at hospital, municipal, and provincial levels to ensure data integrity. Detailed methodological specifications for sampling strategies, data reporting procedures, and quality assurance mechanisms have previously been documented in established protocols (9).

This study was approved by the Ethics Review Committee of Hunan Province Maternal and Children Care Hospital and conducted in accordance with the principles of the Declaration of Helsinki. Because of the retrospective design of this study, the Ethics Review Committee of Hunan Province Maternal and Children Care Hospital has waived the requirement for informed consent for this study.

### Definition of variables

Stillbirth was defined using WHO criteria as fetal deaths occurring at ≥ 28 weeks gestation or with birthweight ≥ 1,000 g, restricted to antepartum cases. To account for conception-to-delivery time lags in policy impact assessment, we operationalized policy exposure windows based on last menstrual period dates (23). The study timeline was stratified into four distinct policy phases: one-child policy (October 2011–September 2013), partial two-child policy (November 2013–November 2015), universal two-child policy (January 2016–December 2020), and universal three-child policy (June 2021–May 2023).

Covariates covering maternal characteristics included: demographics variables such as age (≤24, 25–29, 30–34, ≥35 years) and parity (0, 1, 2, ≥3 prior pregnancies), socioeconomic factors including education level (primary/none, junior high, senior high, college or more) and marital status (married, single/widowed, divorced/cohabiting), attendance at antenatal care visits categorized in line with guidelines (≤5, 6–9, ≥10) and hospital levels classified by geographic proximity to Changsha, the provincial capital(rural, peri-urban, metropolitan).

Obstetric comorbidities were defined as: hypertensive disorders including chronic hypertension (pre-existing or secondary hypertension ± proteinuria), severe preeclampsia (gestational hypertension + proteinuria >0.3 g/24 h or ≥30 mg/mmol) and eclampsia (seizures complicating preeclampsia), endocrine metabolism of preexisting diabetes mellitus and placental pathologies including placenta previa defined as the placenta abnormally covering the endocervical os (30), and placental abruptiondefined as the premature separation of the placenta from its uterine attachment before the delivery of a fetus (31).

### Data selection and sample size

We extracted 917,855 maternal care records from China’s National Maternal Near Miss Surveillance System, spanning October 2011 to May 2023 in Hunan Province. We excluded 71,184 records falling outside the target study period were, giving an initial dataset of 846,671 documented pregnancies. Subsequent application of predefined exclusion criteria sequentially removed multiple gestations (*n* = 18,009), records with missing delivery outcomes (*n* = 83,677), records with incomplete maternal age documentation (*n* = 11,977), pregnancies lacking gestational week documentation (*n* = 3,602), preterm deliveries before 28 weeks’ gestation (*n* = 6,037), and statistical outliers (*n* = 236). Considering that intrapartum stillbirth is a sensitive marker of delay and low quality of care, reflecting scarcity of intrapartum monitoring and delays in the rapid delivery of a compromised fetus, and the 18 Maternal Near Miss Surveillance hospitals quality obstetric and neonatal care is different, the intrapartum stillbirths (*n* = 1,273) were deleted considering that it is a sensitive marker of delay and low quality of care, reflecting scarcity of intrapartum monitoring and delays in the rapid delivery of a compromised fetus but not the health condition of the fetus itself. The final cohort for analysis included 721,860 singleton pregnancies with complete perinatal records, including 5,030 cases of stillbirth. The facility-specific data extraction was listed in Supplementary Table 1.

Based on the following formula for estimating sample size for population incidence rates, the sample size was 6,743 based on the expected incidence rate *p* of 0.46%, allowable absolute error ε less than 0.05%, and width of the 95% confidence interval (CI) is within an acceptable range. The current 721,860 subjects was fully sufficient to meet the sample requirements.

Formula: N $=\frac{z_{1-\alpha /2}^{2}*p*(1-p)}{\varepsilon^{2}}$

### Statistical analysis temporal and stratified analysis

We initially calculated annual stillbirth rates across fertility policy periods (2012–2015: one-child policy; 2016–2020: universal two-child policy; 2021–2023: three-child policy). We used stratified analyses to examine the demographic distribution of stillbirths and live births by maternal characteristics (age, parity, education level, marital status, hospital level, antenatal care frequency) and pregnancy complications across policy epochs. Temporal trends were quantified using segmented regression models.

### Policy period effect estimation

We constructed binary logistic regression to assess unadjusted associations between maternal characteristics and stillbirth risk across policy periods, using the one-child policy era as reference. We also assessed both crude and adjusted associations for pregnancy complications during each policy period, with adjustment for policy exposure duration, maternal age, parity, education attainment, marital status, hospital level and antenatal visit frequency.

### Comprehensive risk profiling

We used integrated models to evaluate population-level stillbirth risks associated with maternal characteristics, adjusting for policy period, demographic factors, and complication status. We also examined complication-specific stillbirth risks, controlling for policy period and demographic covariates.

### Statistical operations

Descriptive statistics were used to characterize baseline maternal profiles. Temporal trends were assessed through segmented regression models with year as the continuous predictor. Multivariable logistic regression generated odds ratios (ORs) with 95% confidence intervals, comparing stillbirths (cases) against live births (controls). All analyses were conducted in R 4.1.0 (R Foundation for Statistical Computing, Vienna, Austria) using two-tailed significance testing (*α* = 0.05).

## Results

### Stillbirth rates across different birth policy periods

**Figure 1:**
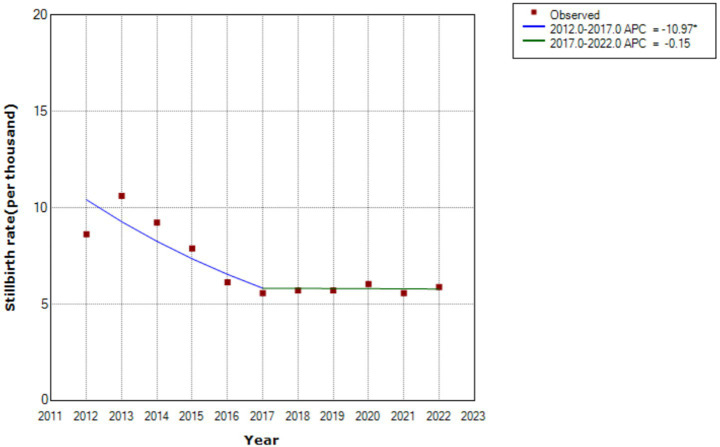
The stillbirth rate during different birth policy periods.

The overall stillbirth rate was 7.02 per 1,000 births (95% CI: 6.83–7.21). However, Table 1 shows, significant temporal variations across policy periods. There was a consistent downward trend from the one-child policy era (9.62‰, 95% CI: 9.08–10.18) to the universal three-child policy phase (5.73‰, 95% CI: 5.25–6.23), with statistically significant linear progression (*t* = −4.22, *p* < 0.01) (Figure 1).

**Table 1:** The stillbirth rate among different birth periods.

Periods	The number of live births	The number of stillbirths	Stillbirth rate (per 1,000 births)	Stillbirths rate and 95% CI
One-child policy period	61,499	530	8.62	9.62 (9.08 10.18)
	61,864	657	10.62	
Partial two-child policy period	70,193	648	9.23	8.54 (8.07 9.03)
	74,288	586	7.89	
Universal two-child policy period	82,763	507	6.13	5.83 (5.59 6.09)
	77,075	429	5.57	
	72,438	414	5.72	
	66,256	379	5.72	
	58,076	351	6.04	
Universal three-child policy period	47,753	266	5.57	5.73 (5.25 6.23)
	44,625	263	5.89	
Total	716,830	5,030	7.02	7.02 (6.82 7.21)

### The stillbirth rate by maternal characteristics under different birth policy periods

**Figure 2:**
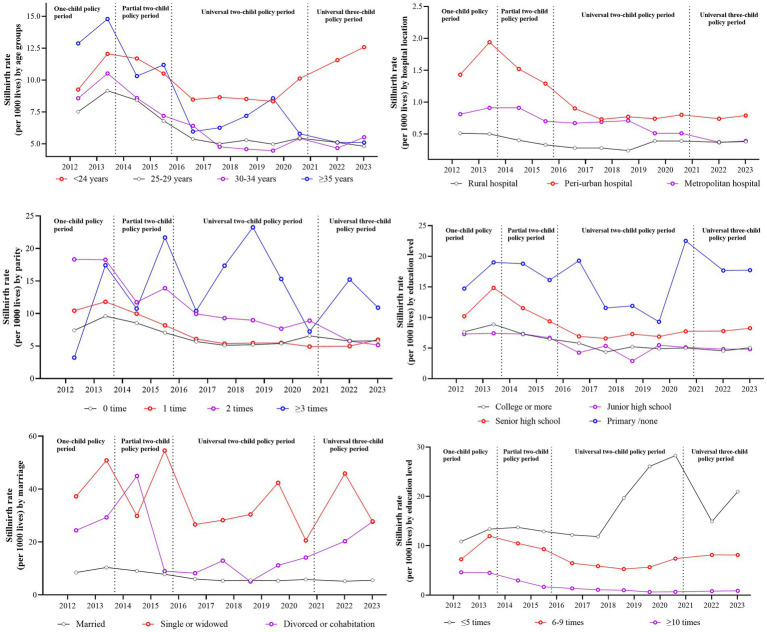
The annual stillbirth incidence by maternal characteristics under different birth policy periods.

Figure 2 shows the annual stillbirth rate by different maternal characteristics under different birth policy periods. Table 2 shows the unadjusted odds ratio of stillbirth rate by different maternal characteristics in different childbirth periods, compared with that in the one-child policy period. The aggregate stillbirth rates were 10.12‰, 6.39‰, 6.03‰ and 7.62‰ for women aged <24, 25–29, 30–34, and ≥35 years. Only those aged < 24 years showed an increasing stillbirth rate during the universal three-child policy period. In contrast, women aged 25–29 years showed no significant temporal decline in stillbirth incidence (*t* = 0.32, *p* = 0.76). Parity analysis showed descending stillbirth rates for pregnancies with no more than two previous pregnancies, while multiparous women (at least one previous pregnancy) maintained consistently higher rates than primiparous women. Lower maternal education levels were correlated with higher stillbirth incidence, showing progressive declines across policy periods except for primary school-educated women. Pregnancies with non-normative marital status were associated with increased stillbirth risks during policy transition phases compared with those during the one-child policy period. The stillbirth rate of pregnancies in different types of hospital gradually decreased with the highest stillbirth rate in hospitals around the provincial capital. Having more antenatal was significantly associated with reduced stillbirth incidence across all policy periods. The frequencies of different maternal characteristics associated with stillbirth across different birth policy periods are shown in Supplementary Table 2.

**Table 2:** The time trend and unadjusted odds ratio of maternal characteristic variables in different childbirth periods compared with one-child policy period.

Variables	One-child policy period	Partial two-child policy period	Universal two-child policy period	Universal three-child policy period	Time trend (t, p)
		OR(95%CI), *p*	OR(95%CI), *p*	OR(95%CI), *p*	
Age (years)					
≤24	Ref	1.06 (0.90 1.24), *p* = 0.50	0.83 (0.72 0.96), *p* = 0.01	1.14 (0.92 1.41), *p* = 0.23	*t* = −4.41, *p* < 0.01
25–29	Ref	0.91 (0.80 1.03), *p* = 0.12	0.62 (0.55 0.70), *p* < 0.01	0.59 (0.49 0.72), *p* < 0.01	*t* = 0.32, *p* = 0.76
30–34	Ref	0.82 (0.69 0.98), *p* = 0.02	0.53 (0.46 0.62), *p* < 0.01	0.53 (0.44 0.64), *p* < 0.01	*t* = −4.30, *p* < 0.01
≥35	Ref	0.78 (0.61 0.99), *p* = 0.04	0.48 (0.40 0.59), *p* < 0.01	0.37 (0.28 0.48), *p* < 0.01	*t* = −4.93, *p* < 0.01
Parity					
0	Ref	0.92 (0.82 1.03), *p* = 0.13	0.66 (0.59 0.72), *p* < 0.01	0.68 (0.59 0.78), *p* < 0.01	*t* = −2.81, *p* = 0.02
1	Ref	0.81 (0.71 0.92), *p* < 0.01	0.50 (0.44 0.56), *p* < 0.01	0.49 (0.41 0.58), *p* < 0.01	*t* = −4.92, *p* < 0.01
2	Ref	0.70 (0.51 0.98), *p* = 0.04	0.48 (0.37 0.64), *p* < 0.01	0.30 (0.21 0.41), *p* < 0.01	*t* = −7.56, *p* < 0.01
≥3	Ref	1.61 (0.62 4.66), *p* = 0.34	1.44 (0.65 3.79), *p* = 0.41	1.29 (0.54 3.57), *p* = 0.59	*t* = 0.29, *p* = 0.78
Hospital location					
Rural	Ref	0.72 (0.60 0.87), *p* < 0.01	0.61 (0.52 0.72), *p* < 0.01	0.74 (0.59 0.94), *p* = 0.01	*t* = −1.29, *p* = 0.27
Peri-urban	Ref	0.83 (0.74 0.92), *p* < 0.01	0.46 (0.42 0.51), *p* < 0.01	0.45 (0.40 0.51), *p* < 0.01	*t* = −4.32, *p* < 0.01
Metropolitan	Ref	0.94 (0.80 1.10), *p* = 0.44	0.73 (0.63 0.85), *p* < 0.01	0.44 (0.34 0.57), *p* < 0.01	*t* = −7.62, *p* < 0.01
Education					
College or more	Ref	0.83 (0.71 0.97), *p* = 0.02	0.61 (0.53 0.70), *p* < 0.01	0.58 (0.49 0.69), *p* < 0.01	*t* = −4.69, *p* < 0.01
Senior high	Ref	0.82 (0.73 0.92), *p* < 0.01	0.55 (0.50 0.61), *p* < 0.01	0.63 (0.54 0.74), *p* < 0.01	*t* = −2.55, *p* = 0.03
Junior high	Ref	0.95 (0.80 0.73), *p* < 0.01	0.62 (0.52 0.73), *p* < 0.01	0.66 (0.49 0.87), *p* < 0.01	*t* = −2.86, *p* = 0.02
Primary/None	Ref	1.04 (0.62 1.77), *p* = 0.88	0.84 (0.51 1.40), *p* = 0.50	1.05 (0.48 2.12), *p* = 0.89	*t* < −0.01, *p* = 0.10
Marriage					
Married	Ref	1.12 (1.03 1.22), *p* = 0.01	0.76 (0.70 0.83), *p* < 0.01	0.86 (0.76 0.97), *p* = 0.02	*t* = −4.56, *p* < 0.01
Single or widowed	Ref	1.47 (0.60 3.48), *p* = 0.39	1.93 (1.05 3.73), *p* = 0.04	2.32 (1.08 5.08), *p* = 0.03	*t* = −0.99, *p* = 0.35
Divorced or cohabitation	Ref	1.25 (0.58 2.61), *p* = 0.56	1.26 (0.67 2.40), *p* = 0.48	4.01 (1.92 8.44), *p* < 0.01	*t* = −0.88, *p* = 0.42
Number of prenatal visits					
≤5 times	Ref	1.30 (1.14 1.49), *p* < 0.01	1.09 (0.96 1.23), *p* < 0.18	1.17 (0.95 1.45), *p* = 0.14	*t* = 2.71, *p* = 0.02
6–9 times	Ref	1.06 (0.94 1.20), *p* = 0.33	0.74 (0.66 0.83), *p* < 0.01	0.89 (0.76 1.04), *p* = 0.13	*t* = −1.35, *p* = 0.21
≥10 times	Ref	0.51 (0.38 0.69), *p* < 0.01	0.21 (0.16 0.29), *p* < 0.01	0.20 (0.13 0.31), *p* < 0.01	*t* = −5.10, *p* < 0.01

### The stillbirth rate by maternal complications across birth policy periods

**Figure 3:**
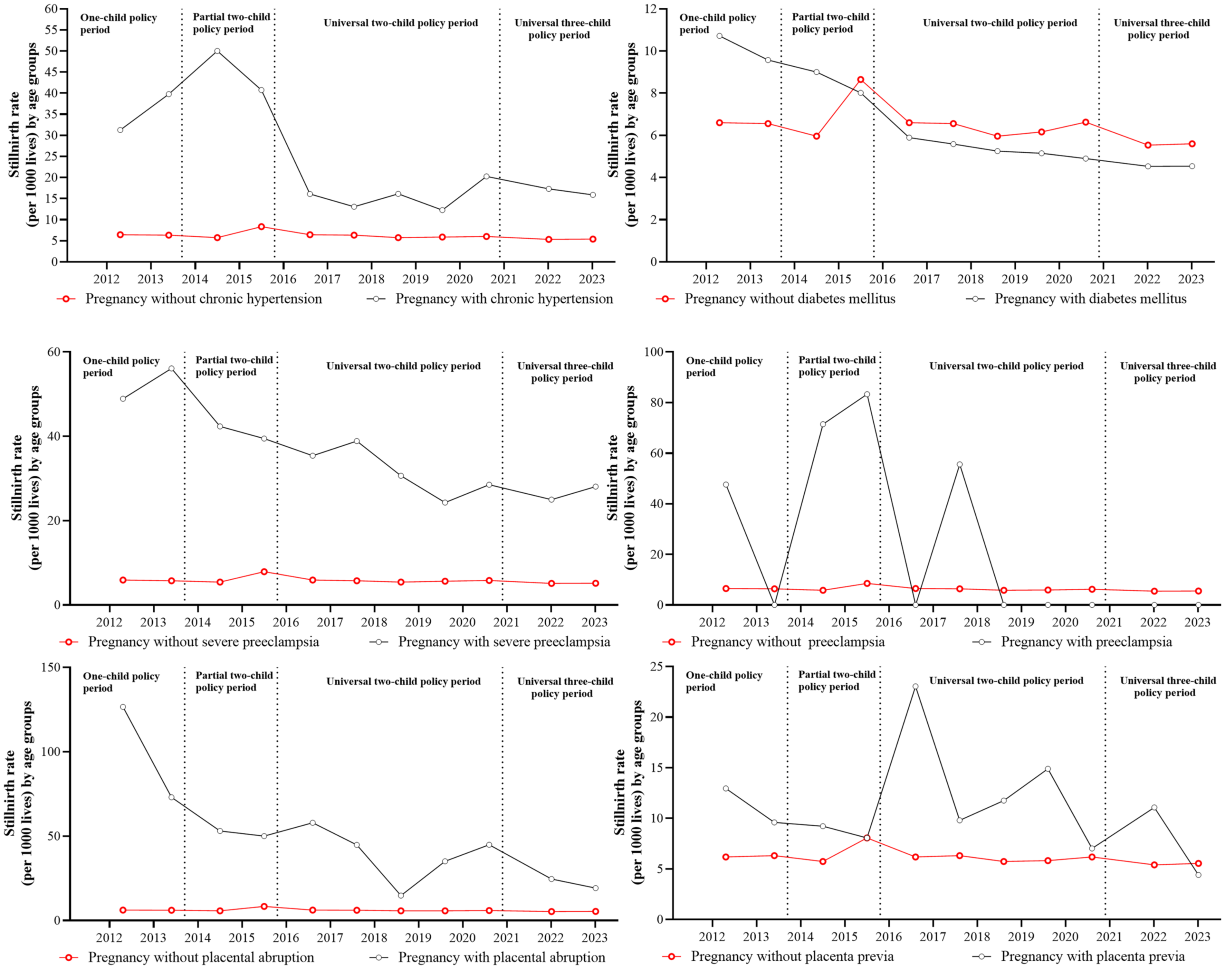
The incidence and related trends of stillbirths by complications during different birth policy periods.

Figure 3 shows annual stillbirth rates stratified by maternal complications under different birth policy periods. Table 3 shows unadjusted and adjusted odds ratios for stillbirth risk across these policy periods, and Supplementary Table 3 shows the frequency distribution of pregnancies with complications and stillbirths across policy phases. Within the study cohort of 458,636 pregnancies with complications (63.54%), the prevalence of specific complications was as follows: prenatal diabetes mellitus (15.85%, *N* = 72,705), severe preeclampsia (2.07%, *N* = 9,471), placenta previa (1.92%, *N* = 8,810), chronic hypertension (0.84%, *N* = 3,858), placental abruption (0.61%, *N* = 2,775), and eclampsia (0.03%, *N* = 153). Comparative analysis showed significantly higher stillbirth rates in pregnancies complicated by prenatal hypertension, severe preeclampsia, placenta previa, and placental abruption compared with the rates in uncomplicated pregnancies across all policy periods.

**Table 3:** The unadjusted and adjusted odds ratio of different maternal near misses in each child policy period.

Variables	One-child policy period		Partial two-child policy period		Universal two-child policy period		Universal three-child policy period	
	Unadjust OR (95%CI)	Adjust OR (95%CI)*	Unadjust OR (95%CI)	Adjust OR (95%CI)*	Unadjust OR (95%CI)	Adjust OR (95%CI)*	Unadjust OR (95%CI)	Adjust OR (95%CI)*
Severe preeclampsia								
No	Ref	Ref	Ref	Ref	Ref	Ref	Ref	Ref
Yes	5.40 (4.22, 6.83)	4.02 (3.00, 5.32)	4.9 (3.78, 6.25)	2.85 (2.14, 3.74)	5.62 (4.70, 6.67)	4.36 (3.62, 5.22)	5.16 (3.61, 7.16)	3.27 (2.24, 4.65)
Eclampsia								
No	Ref	Ref	Ref	Ref	Ref	Ref	Ref	Ref
Yes	2.77 (0.45, 8.88)	0.97 (0.06, 4.54)	8.56 (1.38, 28.7)	2.37 (0.13, 12.5)	3.12 (0.18, 14.20)	1.38 (0.08, 6.58)	0 (0.00, 0.00)	0 (0.00, 0.00)
Pregnancy with chronic hypertension								
No	Ref	Ref	Ref	Ref	Ref	Ref	Ref	Ref
Yes	3.25 (1.67, 5.69)	2.23 (0.99, 4.37)	5.04 (3.10, 7.72)	2.77 (1.55, 4.60)	2.62 (1.81, 3.67)	2.66 (1.82, 3.75)	3.10 (1.83, 4.89)	2.45 (1.40, 3.99)
Diabetes mellitus								
No	Ref	Ref	Ref	Ref	Ref	Ref	Ref	Ref
Yes	0.87 (0.66, 1.12)	0.67 (0.42, 1.03)	0.93 (0.75, 1.14)	0.79 (2.34, 6.04)	0.83 (0.73, 0.95)	0.95 (0.82, 1.08)	0.81 (0.52, 1.21)	0.67 (0.42, 1.03)
Placental abruption								
No	Ref	Ref	Ref	Ref	Ref	Ref	Ref	Ref
Yes	8.26 (5.20, 12.50)	6.87 (3.93, 11.30)	5.88 (3.74, 8.78)	3.86 (2.34, 6.04)	6.44 (4.88, 8.34)	5.68 (4.26, 7.43)	4.09 (2.03, 7.30)	3.53 (1.73, 6.42)
Placenta previa								
No	Ref	Ref	Ref	Ref	Ref	Ref	Ref	Ref
Yes	1.95 (1.34, 2.75)	1.61 (1.04, 2.38)	2.71 (2.00, 3.57)	1.63 (1.18, 2.19)	2.27 (1.71, 2.95)	1.65 (1.22, 2.18)	1.47 (0.67, 2.76)	0.93 (0.42, 1.78)

*The adjust variables were maternal characteristic variables including birth policy periods, age, parity, hospital, education level, marriage status, and number of prenatal visits.

Two complications showed consistent associations with elevated stillbirth risk across all policy periods: severe preeclampsia (adjusted OR range: 2.85–4.36) and placental abruption (adjusted OR range: 3.53–6.87). Hypertension-related complications and placenta previa both showed policy-dependent risk patterns, with non-significant associations observed specifically during the one-child policy and universal three-child policy periods.

### Risk factors associated with stillbirth across birth policy periods

The unadjusted and adjusted odds ratio for stillbirth associated with maternal characteristics and six pregnancy complications across all the birth policy periods are shown in Table 4. Binary logistic regression analysis showed several significant risk factors for stillbirth after adjustment for confounding variables. These included exposure to the partial two-child policy period, maternal age ≤24 years (aOR = 1.77, 95%CI: 1.63–1.92), multiparity (aOR = 1.27–2.82), non-rural hospital location (aOR = 4.00–11.13), educational attainment below university level, being unmarried, and the presence of severe preeclampsia, prenatal hypertension, placental abruption, or placenta previa. There were also two protective factors: pregnancies complicated by diabetes mellitus (aOR = 0.86, 95%CI: 0.77–0.95) and receiving more frequent prenatal check-ups (aOR = 0.06–0.34) were associated with lower stillbirth risk.

**Table 4:** The total unadjusted and adjusted odds ratio of stillbirth among maternal characteristics and different maternal near misses.

Variables	Stillbirth			
	Crude odds ratio (95%CI)	*p*	Adjusted odds ratio (95%CI)	*p*
Birth policy periods				
One-child policy period	Ref		Ref	
Partial two-child policy period	0.89 (0.82, 0.96)	<0.01	1.15(1.05, 1.25)^*^	<0.01
Universal two-child policy period	0.61 (0.56, 0.65)	<0.01	0.79(0.73, 0.86)^*^	<0.01
Universal three-child policy period	0.60 (0.54, 0.66)	<0.01	0.93(0.83, 1.05)^*^	0.23
Age (years)				
25–29	Ref		Ref	
≤24	1.58 (1.47, 1.71)	<0.01	1.77(1.63, 1.92)^*^	<0.01
30-34	0.94 (0.88, 1.01)	0.11	0.86(0.80, 0.93)^*^	<0.01
≥35	1.19 (1.09, 1.30)	<0.01	0.91(0.83, 0.10)^*^	<0.01
Parity				
0	Ref		Ref	
1	1.02 (0.96, 1.08)	0.51	1.27(1.18, 1.35)^*^	<0.01
2	1.41 (1.27, 1.57)	<0.01	2.02(1.79, 2.27)^*^	<0.01
≥3	2.05 (1.62, 2.58)	<0.01	2.82(2.18, 3.60)^*^	<0.01
Hospital				
Rural	Ref		Ref	
Peri-urban	2.75 (2.56, 2.96)	<0.01	11.13(10.12, 12.26)^*^	<0.01
Metropolitan	1.86 (1.71, 2.03)	<0.01	4.00(3.63, 4.41)^*^	<0.01
Education				
College or more	Ref		Ref	
Senior high school	1.55 (1.45, 1.65)	<0.01	1.73(1.62, 1.86)^*^	<0.01
Junior high school	1.05 (0.96, 1.14)	0.28	1.51(1.35, 1.68)^*^	<0.01
Primary/none	2.83 (2.32, 3.47)	<0.01	2.20(1.72, 2.79)^*^	<0.01
Marriage				
Married	Ref		Ref	
Single or widowed	4.83 (4.04, 5.78)	<0.01	5.60(4.62, 6.74)^*^	<0.01
Divorced or cohabitation	2.47 (2.00, 3.06)	<0.01	2.92(2.31, 3.65)^*^	<0.01
Number of prenatal visits				
≤5 times	Ref		Ref	
6–9 times	0.53 (0.50, 0.56)	<0.01	0.34(0.32, 0.36)^*^	<0.01
≥10 times	0.10 (0.09, 0.11)	<0.01	0.06(0.05, 0.06)^*^	<0.01
Severe preeclampsia				
No	Ref		Ref	
Yes	5.64 (5.02, 6.33)	<0.01	3.80(3.36, 4.29)^**^	<0.01
Eclampsia				
No	Ref		Ref	
Yes	4.79 (1.96, 11.68)	<0.01	1.62(0.49, 3.96)^**^	0.35
Pregnancy with chronic hypertension				
No	Ref		Ref	
Yes	3.05 (2.44, 3.81)	<0.01	2.67 (2.09, 3.37)^**^	<0.01
Diabetes mellitus				
No	Ref		Ref	
Yes	0.86 (0.78, 0.95)	<0.01	0.86 (0.77, 0.95)^**^	<0.01
Placental abruption				
No	Ref		Ref	
Yes	6.25 (5.17, 7.56)	<0.01	5.06 (4.11, 6.16)^**^	<0.01
Placenta previa				
No	Ref		Ref	
Yes	2.45 (2.07, 2.90)	<0.01	1.55 (1.29, 1.84)^**^	<0.01

*The adjust variables were birth policy periods, age groups, parity, hospital, education level, marriage status, number of prenatal visits and complications occurrence.

**The adjust variables were the maternal near miss and maternal characteristic variables including birth policy periods, age, parity, hospital, education level, marriage status, and number of prenatal visits.

## Discussion

This longitudinal policy-sensitive analysis shows critical patterns in the epidemiology of stillbirth across changes in China’s fertility policy form 2011 to 2024. We found an overall stillbirth rate of 7.02 per 1,000 live births (95%CI 6.82–7.21), with a significant annual decline of 4.82% and a J-shaped association with stillbirth risk, with the lowest rate among women aged 25–29 years. Those aged < 24 years showed a paradoxical increase in stillbirth rate after the COVID-19 pandemic. Multiparity, educational attainment of ≤9 years, being unmarried, delivery in a secondary hospital, attending less than five antenatal care appointments, preconception hypertension, severe preeclampsia, placenta accreta, and placenta previa were all risk factors of stillbirths. Pre-existing diabetes mellitus showed a paradoxical risk, with diabetic mothers showing lower stillbirth incidence than non-diabetic controls during the universal two−/three-child policy periods.

The stillbirth rate of Hunan province in the Central region of China, was higher than that in South, and lower than that in the Northwest, highlighting the uneven distribution of stillbirths across geographical regions in China (8). The stillbirth rate was also higher than that reported (4.5 per 1,000 births from January 2010 to December 2019 in Baoan, Shenzhen) (10), and lower than that the average of 8.8 per 1,000 births reported for China during 2012 to 2014 (9). Our stillbirth rate was also higher than the 5.8 per 1,000 total births at ≥24 weeks in the United states in 2020 (32), and 0.09 per 1,000 births of term intrapartum stillbirths in Norway from 1999 to 2018 (33). However, it was lower than the global stillbirth rate of 13.9 per 1,000 total births at or beyond 28 weeks of gestation (34), and the rate for most other low and middle-income countries (12), such as India (35). The average annual reduced rate was the same as the national average of 4–6% (36). The decline in rate of stillbirth was greatest (37%) from the partial two-child policy period to the universal two-child policy period. After that, it continued to decrease slightly. This phenomenon may be related to fertility intention and the total numbers of live births. After the enactment of China’s new universal two- child policy, the change in fertility intention among Chinese women (intention to have a second child) was short-term (19). The total number of births only increased during the partial two-child policy period (25). This study therefore found that only the partial two-child policy period was associated with the risk of stillbirth, which can be attributed to factors such as a higher proportion of pregnant women over 35 years, an increase in pregnancy complications and comorbidities, and potential impacts on obstetric service capabilities (37–39).

The prevention and reduction of stillbirths require targeted interventions for key risk groups, particularly pregnant women under 24 and over 35 years of age. Additional support is also needed for multiparous women, and those with limited education, or who are unmarried, delivering in rural hospitals, and have fewer than five antenatal care visits. Our analysis uncovered a paradoxical age-specific pattern in stillbirth rates following China’s evolving fertility policy framework. Women aged under 24 years had the highest stillbirth incidence across all age groups following the implementation of the 2016 universal two-child policy. This trend intensified post-2021, when the three-child policy started, establishing this group as the sole demographic exhibiting with rising stillbirth rates during policy transitions. This epidemiological pattern aligns with established risk profiles for stillbirth, such as being young, unmarried, and with low educational attainment (9). These risk factors predominantly cluster in those under 24 years, particularly given the diminished fertility intentions among women of optimal childbearing age since the implementation of the three-child policy (40). These findings underscore the need for focused stillbirth prevention strategies targeting women under 24, including enhanced contraceptive education and access (9, 41). Contrastingly, the period before 2016 showed higher stillbirth risk among older women, attributable to both inherent biological risks (5, 8) and fertility demand release during the partial two-child policy phase. We also found parity-dependent risk escalation, consistent with perinatal outcome studies (28, 42). This association may reflect the dual mechanisms of older women being more likely to have other children and early pregnancies occurring in socioeconomically disadvantaged contexts where there is limited health literacy.

This study identified placenta accreta and placenta previa among placental factors, and severe preeclampsia and prenatal hypertension among hypertensive disorders, as significant risk factors for stillbirth. These findings are consistent with other evidence showing elevated stillbirth rates in pregnancies with maternal comorbidities. For instance, singleton births in Gambian hospitals from January to June 2006 showed an eight-fold increase in risk of stillbirth among women with severe obstetric complications compared with the risk among those without complications (43, 44). Hypertensive disorders in pregnancy are categorized into four major subtypes: pregnancy-induced hypertension (PIH, mild to moderate hypertension without proteinuria), preeclampsia (hypertension with proteinuria), severe preeclampsia, and eclampsia (45). Hypertension during pregnancy is widely recognized as a risk factor for stillbirth, but our analysis showed no significant association between eclampsia and stillbirth, potentially attributable to pharmacological interventions during eclamptic episodes. Emerging evidence suggests that calcium supplementation during pregnancy may reduce stillbirths by 19%, though this finding was not statistically significant, while aspirin use showed no protective association (46). Future investigations should explore mediating factors between hypertensive disorders and stillbirth pathogenesis.

As China’s maternal medical care system has improved, general gestational health has also improved and there have been better outcomes among infants, as well as lower incidence of hypertensive disorders in pregnancy and placenta previa after the introduction of the universal two-child policy in one of our monitoring hospitals (20). Our results, together with others, suggest that placental factors may contribute to stillbirth (47–49). Nationwide studies also showed placenta accreta was a critical risk factor for antepartum stillbirth in central China (8). However, contrary to conventional risk paradigms, prenatal diabetes was not associated with increased stillbirth risk in our study. Notably, since the implementation of the universal two-child policy in China, diabetic pregnancies have resulted in a lower incidence of stillbirth than non-diabetic pregnancies. This observation is consistent with studies identifying maternal blood glucose levels and body mass index as modifiable risk factors for stillbirth in diabetic pregnancies (50). Future investigations should explore mediating factors between prenatal diabetes and stillbirth.

It is still unclear whether maternal comorbidities precipitate stillbirths or vice versa. We had no data on prior history of stillbirth, but cohort data from Western Australian from 2000 to 2015 show that mothers who have stillbirths, regardless of any comorbidities have a great risk of severe maternal morbiditythan mothers who have live births (48). Future research should prioritize understanding the temporal relationship between comorbidities and fetal death (51). Healthcare quality improvements, socioeconomic changes during China’s rapid development, Body Mass Index, smoking status, previous obstetric history, COVID-19 pandemic impact overlaps with policy influence should be taken account for analysis of factors influencing stillbirths.

Our study is important for several reasons. First, it is the first study of which we are aware to characterize stillbirth patterns and identify influencing factors across different policy periods. Second, our research framework incorporated a critical assessment of policy lag effects, a temporal dimension frequently overlooked in epidemiological studies examining the impact of fertility policies. Third, the investigation covered 18 surveillance hospitals across the province, ensuring a large sample size that enhances the statistical power of our findings.

However, the study also had several limitations. First, it was a cross-sectional retrospective investigation, and the observed associations between risk factors and stillbirth therefore require validation through long-term longitudinal observations and prospective cohort studies. Second, hospital selection followed the urban–rural distribution patterns from the 2010 national census to ensure national representativeness, but this sampling strategy may not fully reflect the specific demographic characteristics of Hunan Province. What’s more, this hospital-based sampling data may not represent the whole province population-level estimates for the selection bias and generalizability limitations. Third, the dataset lacked detailed clinical parameters such as maternal anthropometrics (weight/height) and obstetrical history indicators (previous occurrence of stillbirth), which could potentially influence the analysis outcomes.

## Conclusion

We found that the selective two-child policy, maternal age of <24 years, multiparity, non-rural hospital delivery, lower levels of education, inadequate antenatal care, and comorbidities including prenatal hypertension, severe preeclampsia, placenta accreta, and placenta previa were significantly associated with elevated risk of stillbirth. Temporal trend analysis showed distinct patterns: advanced maternal age was linked to higher stillbirth rates before the two-child policy liberalization. However, post-liberalization, younger mothers (age <24 years) had higher incidence of stillbirth. Pregnancies complicated by prenatal diabetes actually showed a lower risk of stillbirth than non-diabetic pregnancies. Future preventive strategies should prioritize high-risk subgroups, including younger mothers, those with lower educational attainment, women with other children, and pregnancies complicated by hypertensive disorders or placental pathologies. This should help to mitigate stillbirths. As national efforts move from reducing infant mortality to enhancing perinatal care, together with the promotion of fertity after the implementation of China’s three-child policy, addressing stillbirth remains a critical clinical and public health priority. Integrating stillbirth metrics into maternal and neonatal care quality surveillance and enhancing perinatal care accessibility remain urgent public health priorities.

## Data Availability

The original contributions presented in the study are included in the article/Supplementary material, further inquiries can be directed to the corresponding authors.
