# Supplementary material for: The incidence, characteristics, and complications of pregnant women who delivered stillbirths under different child policies in central China
Source: Front Public Health. 2025 Oct 7;13:1635120. doi: 10.3389/fpubh.2025.1635120 (PMC12537710; doi:10.3389/fpubh.2025.1635120)
Supplement: Supplementary file 1 [file Table_1.docx]

Plus-table 1. The final data analysis entered into the database for each hospital.

| Hospital | Location | The initial number of cases | Number of cases included in the analysis |
| --- | --- | --- | --- |
| Hunan Provincial Maternal and Child Health Care Hospital | Metropolitan | 164133 | 147027 |
| Changsha city Maternal and Child Health Hospital | Metropolitan | 139678 | 123922 |
| The Second Xiangya Hospital of Central South University | Metropolitan | 25970 | 20624 |
| Liuyang City Maternal and Child Health Care Hospital | Peri-urban | 83887 | 73414 |
| Liuyang City People's Hospital | Peri-urban | 37606 | 32077 |
| Zhuzhou City Maternal and Child Health Hospital | Peri-urban | 32556 | 26558 |
| Zhuzhou City Central Hospital | Peri-urban | 45909 | 38740 |
| Anhua County Maternal and Child Health Care Hospital | Rural | 11517 | 8716 |
| Anxiang County Maternal and Child Health Care Hospital | Rural | 17791 | 14363 |
| Guiyang County People's Hospital | Rural | 25914 | 21416 |
| Lengyang City Maternal and Child Health Care Hospital | Rural | 41165 | 35023 |
| Leiyang City People's Hospital | Rural | 41831 | 34947 |
| Pingjiang County First People's Hospital | Rural | 55316 | 47042 |
| Pingjiang County Maternal and Child Health Care Hospital | Rural | 37942 | 28611 |
| Xiangxiang City Maternal and Child Health Care Hospital | Rural | 30332 | 25443 |
| Xiangxiang City People's Hospital | Rural | 34325 | 28705 |
| Xintian County People's Hospital | Rural | 12168 | 8895 |
| Yongshun County Maternal and Child Health Care Hospital | Rural | 8631 | 6337 |
| Total | 18 | 846671 | 721860 |
