# Supplementary material for: The incidence, characteristics, and complications of pregnant women who delivered stillbirths under different child policies in central China
Source: Front Public Health. 2025 Oct 7;13:1635120. doi: 10.3389/fpubh.2025.1635120 (PMC12537710; doi:10.3389/fpubh.2025.1635120)
Supplement: Supplementary file 2 [file Table_2.docx]

Plus-table 2. The number of the characterics of mothers who delived the stillbirths among different birth policy periods in Hunan province,China.

| Variables | One-child policy period | | Partial two-child policy period | | Universal two-child policy period | | Universal three-child policy period | | Total | |
| --- | --- | --- | --- | --- | --- | --- | --- | --- | --- | --- |
|  | Live births | Stillbirths | Live births | Stillbirths | Live births | Stillbirths | Live births | Stillbirths | Live births | Stillbirths |
|  | (N=123363,99.0%) | (N=1187,1.0%) | (N=144481,99.2%) | (N=1234,0.8%) | (N=356608,99.4%) | (N=2080,0.6%) | (N=92378, 99.4%) | (N=529, 0.6%) | (N=92378, 99.4%) | (N=529,0.6%) |
| **Age(years)** | | | | | | | | | | |
| 25-29 | 55470 (99.2) | 464 (0.8) | 71908 (99.2) | 545 (0.8) | 131659 (99.5) | 686 (0.5) | 27401 (99.5) | 136 (0.5) | 286438 (40.0) | 1831 (36.4) |
| ≤24 | 33800 (99.0) | 357 (1.0) | 23664 (98.9) | 264 (1.1) | 41901 (99.1) | 367 (0.9) | 9051 (98.8) | 109 (1.2) | 108416 (15.1) | 1097 (21.8) |
| 30-34 | 24866 (99.1) | 238 (0.9) | 34727 (99.2) | 272 (0.8) | 124932 (99.5) | 638 (0.5) | 36339 (99.5) | 184 (0.5) | 220864 (30.8) | 1332 (26.5) |
| ≥35 | 9227 (98.6) | 128 (1.4) | 14182 (98.9) | 153 (1.1) | 58116 (99.3) | 389 (0.7) | 19587 (99.5) | 100 (0.5) | 101112 (14.1) | 770 (15.3) |
| **Parity** | | | | | | | | | | |
| 0 | 80644 (99.2) | 685 (0.8) | 78970 (99.2) | 617 (0.8) | 155292 (99.4) | 864 (0.6) | 44607 (99.4) | 258 (0.6) | 359513 (50.2) | 2424(48.2) |
| 1 | 38405 (98.9) | 428 (1.1) | 59107 (99.1) | 532 (0.9) | 174990 (99.5) | 967 (0.5) | 37413 (99.5) | 204 (0.5) | 309915 (43.2) | 2131 (42.4) |
| 2 | 3716 (98.2) | 68 (1.8) | 5663 (98.7) | 73 (1.3) | 23618 (99.1) | 210 (0.9) | 8967 (99.5) | 49 (0.5) | 41964 (5.9) | 400 (8.0) |
| ≥3 | 598 (99.0) | 6 (1.0) | 741 (98.4) | 12 (1.6) | 2708 (98.6) | 39 (1.4) | 1391 (98.7) | 18 (1.3) | 5438 (0.8) | 75 (1.5) |
| **Hospital** | | | | | | | | | | |
| Rural | 52658 (99.5) | 266 (0.5) | 54213 (99.6) | 198 (0.4) | 125624 (99.7) | 388 (0.3) | 26053 (99.6) | 98 (0.4) | 258548 (36.1) | 950 (18.9) |
| Peri-urban | 37639 (98.3) | 637 (1.7) | 52060 (98.6) | 728 (1.4) | 152527 (99.2) | 1200 (0.8) | 46427 (99.2) | 355 (0.8) | 288653 (40.3) | 2920(58.1) |
| Metropolitan | 33066 (99.1) | 284 (0.9) | 38208 (99.2) | 308 (0.8) | 78457 (99.4) | 492 (0.6) | 19898 (99.6) | 76 (0.4) | 169629 (23.7) | 1160 (23.1) |
| **Education level** | | | | | | | | | | |
| College or more | 33164 (99.2) | 273 (0.8) | 47658 (99.3) | 326 (0.7) | 150190 (99.5) | 757 (0.5) | 55820 (99.5) | 268 (0.5) | 286832 (40.3) | 1624 (32.6) |
| Senior high | 46998 (98.8) | 593 (1.2) | 58983 (99.0) | 612 (1.0) | 146475 (99.3) | 1026 (0.7) | 24569 (99.2) | 196 (0.8) | 277025 (39.0) | 2427 (48.6) |
| Junior high | 37823 (99.3) | 278 (0.7) | 34627 (99.3) | 243 (0.7) | 57137 (99.5) | 260 (0.5) | 11415 (99.5) | 55 (0.5) | 141002 (19.8) | 836 (16.8) |
| Primary/None | 1604 (98.3) | 27 (1.7) | 1712 (98.3) | 30 (1.7) | 2475 (98.6) | 35 (1.4) | 565 (98.3) | 10 (1.7) | 6356 (0.9) | 102 (2.0)\| |
| **Marriage status** | | | | | | | | | | |
| Married | 122192 (99.1) | 1149 (0.9) | 143525 (99.2) | 1208 (0.8) | 351162 (99.4) | 1973 (0.6) | 90858 (99.5) | 485 (0.5) | 707737 (98.7) | 4815 (95.7) |
| Single or widowed | 365 (95.8) | 16 (4.2) | 244 (96.1) | 10 (3.9) | 2628 (97.1) | 78 (2.9) | 629 (96.5) | 23 (3.5) | 3866 (0.5) | 127 (2.5) |
| Divorced or cohabitation | 806 (97.3) | 22 (2.7) | 712 (97.8) | 16 (2.2) | 2818 (99.0) | 29 (1.0) | 891 (97.7) | 21 (2.3) | 5227 (0.7) | 88 (1.7) |
| **Number of prenatal visits** | | | | | | | | | | |
| ≤5 times | 43305 (98.8) | 516 (1.2) | 33492 (98.7) | 446 (1.3) | 54869 (98.3) | 922 (1.7) | 7301 (98.3) | 128 (1.7) | 138967 (19.8) | 2012 (41.2) |
| 6-9 times | 50674 (99.0) | 494 (1.0) | 65083 (99.0) | 643 (1.0) | 167454 (99.4) | 1021 (0.6) | 44795 (99.2) | 365 (0.8) | 328006 (46.8) | 2523 (51.7) |
| ≥10 times | 22092 (99.5) | 101 (0.5) | 38857 (99.8) | 88 (0.2) | 132083 (99.9) | 123 (0.1) | 40161 (99.9) | 34 (0.1) | 233193 (33.3) | 346 (7.1) |
