# Supplementary material for: The incidence, characteristics, and complications of pregnant women who delivered stillbirths under different child policies in central China
Source: Front Public Health. 2025 Oct 7;13:1635120. doi: 10.3389/fpubh.2025.1635120 (PMC12537710; doi:10.3389/fpubh.2025.1635120)
Supplement: Supplementary file 3 [file Table_3.docx]

Plus-table 3. The number of the different maternal near miss among mothers who delivered stillbirths among different birth policy periods in Hunan province, China.

| Variables | One-child policy period | | Partial two-child policy period | | Universal two-child policy period | | Universal three-child policy period | | Total | |
| --- | --- | --- | --- | --- | --- | --- | --- | --- | --- | --- |
|  | Live births | Stillbirths | Live births | Stillbirths | Live births | Stillbirths | Live births | Stillbirths | Live births | Stillbirths |
|  | (N=44105,98.87%) | (N=498,1.13%) | (N=82036,99.10%) | (N=739,0.90%) | (N=250807,99.38%) | (N=1548,0.62%) | (N=78435,99.45%) | (N=432,0.55%) | (N=78435,99.45%) | (N=432,0.55%) |
| Severe preeclampsia | | | | | | | | | | |
| No | 42508(99.0) | 414(1.0) | 80321(99.2) | 669(0.8) | 246379(99.4) | 1406(0.6) | 77073(99.5) | 395(0.5) | 446281 (98.0) | 2884 (89.6) |
| Yes | 1597(95.0) | 84(5.0) | 1715(96.1) | 70(3.9) | 4428(96.9) | 142(3.1) | 1398(97.4) | 37(2.6) | 9138 (2.0) | 333 (10.4) |
| Eclampsia | | | | | | | | | | |
| No | 44041(98.9) | 496(1.1) | 82010(99.1) | 737(0.9) | 250755(99.4) | 1547(0.6) | 78465(99.5) | 432(0.5) | 455271 (100.0) | 3212 (99.8) |
| Yes | 64(97.0) | 2(3.0) | 26(92.9) | 2(7.1) | 52(98.1) | 1(1.9) | 6(100.0) | 0(0.0) | 148 (0.0) | 5 (0.2) |
| Pregnancy with chronic hypertension | | | | | | | | | | |
| No | 43800(98.9) | 487(1.1) | 81586(99.1) | 719(0.9) | 248806(99.4) | 1516(0.6) | 77448(99.5) | 415(0.5) | 451640 (99.2) | 3137 (97.5) |
| Yes | 304(96.5) | 11(3.5) | 450(95.7) | 20(4.3) | 2001(98.4) | 32(1.6) | 1023(98.4) | 17(1.6) | 3778 (0.8) | 80 (2.5) |
| Placental abruption | | | | | | | | | | |
| No | 43848(98.9) | 475(1.1) | 81590(99.1) | 716(0.9) | 249301(99.4) | 1490(0.6) | 78019(99.5) | 422(0.5) | 452758 (99.4) | 3103 (96.5) |
| Yes | 257(91.8) | 23(8.2) | 446(95.1) | 23(4.9) | 1506(96.3) | 58(3.7) | 452(97.8) | 10(2.2) | 2661 (0.6) | 114 (3.5) |
| Diabetes mellitus | | | | | | | | | | |
| No | 37883(98.9) | 436(1.1) | 69758(99.1) | 635(0.9) | 202126(99.4) | 1289(0.6) | 73395(99.4) | 409(0.6) | 383162 (84.1) | 2769 (86.1) |
| Yes | 6222(99.0) | 62(1.0) | 12278(99.2) | 104(0.8) | 48681(99.5) | 259(0.5) | 5076(99.5) | 23(0.5) | 72257 (15.9) | 448 (13.9) |
| Placenta previa | | | | | | | | | | |
| No | 42560(98.9) | 465(1.1) | 79847(99.1) | 688(0.9) | 246872(99.4) | 1494(0.6) | 77475(99.5) | 424(0.5) | 446754 (98.1) | 3071 (95.5) |
| Yes | 1545(97.9) | 33(2.1) | 2188(97.7) | 51(2.3) | 3935(98.6) | 54(1.4) | 996(99.2) | 8(0.8) | 8664 (1.9) | 146 (4.5) |
